# The causal mediating effect of smoking on the relationship between irritability and bipolar disorder: A two-step Mendelian randomization study

**DOI:** 10.18332/tid/209615

**Published:** 2025-11-07

**Authors:** Qianying Hu, Chaoyan Yue, Yifeng Xu, Jianhua Chen, Xin Luo, Enzhao Cong

**Affiliations:** 1 Shanghai Tenth People's Hospital, School of Medicine, Tongji University, Shanghai, China; 2 Shanghai Mental Health Center, Shanghai Jiao Tong University School of Medicine, Shanghai, China; 3 Obstetrics and Gynecology Hospital of Fudan University, Shanghai, China

**Keywords:** Mendelian randomization, irritability, smoking, bipolar disorder, mediation

## Abstract

**INTRODUCTION:**

Bipolar disorder is a periodic episode of extreme fluctuations in emotion that has been shown to be associated with smoking and irritability, but the relationship between the three has not been studied, especially in terms of genetic causality. This study aimed to obtain potential causal estimates of the association between irritability and bipolar disorder while quantifying the mediating effects of the modifiable risk factor, smoking.

**METHODS:**

This study used a two-step Mendelian randomization (MR) method and employed the inverse variance weighted method for the two-sample MR, utilizing SNPs as genetic instruments. Sensitivity analyses were conducted to detect heterogeneity and horizontal pleiotropy.

**RESULTS:**

Irritability (OR=3.13; 95% CI: 1.23–7.93; p=0.016) and smoking (OR=2.00; 95% CI: 1.47–2.37; p<0.001) were significantly associated with bipolar disorder from a genetic perspective. Irritability was associated with a higher risk of smoking (OR=1.21; 95% CI: 1.07–1.37; p=0.002). Sensitivity analyses confirmed the robustness of these results. Mediation analysis indicated that smoking partially mediated the potential pathway from irritability and bipolar disorder, with the proportion of the effect of irritability on bipolar disorder mediated by smoking being 11.76% (95% CI: 2–21; p=0.012).

**CONCLUSIONS:**

Smoking plays a mediating role in the potential causal pathway linking irritability and bipolar disorder, suggesting that smoking cessation interventions may possibly help mitigate the risk of bipolar disorder among individuals with heightened irritability.

## INTRODUCTION

Bipolar disorder is characterized by the cyclical occurrence of manic episodes and major depressive episodes, resulting in significant distress and impairment in daily social functioning according to DSM-5. The global prevalence of bipolar disorder is estimated to be 0.5% and the associated disability-adjusted life years are 67^1^. Patients with bipolar disorder exhibit significant cognitive impairments overall^2^. Compared to healthy individuals, their cognitive deficits lay in language learning, verbal memory, working memory, visual learning, and visual memory^3^. Additionally, bipolar disorder is often associated with increased suicidal ideation and attempts, and potentially extended hospitalizations for intensive care^4^. Clinically, bipolar disorder often initially presents with a major depressive episode, with its defining feature, mania, only emerging after months or years, resulting in considerable delays^5^. During this period, patients may be misdiagnosed with depression and treated with antidepressants, which can worsen mixed or manic symptoms, or induce severe mood instability^6^. This phenomenon is relatively common, highlighting the importance of early and accurate differentiation of bipolar disorder as a critical clinical issue.

Irritability is considered a significant symptom of bipolar disorder, referring to an individual’s tendency to become easily angry by external stimuli. Compared with peers, it manifests as temper outbursts and mood swings that are disproportionate to the developmental stage or situational context^7^. Studies show that 80% of adolescents with bipolar disorder exhibit irritability, which is considered a ‘broad phenotype’, while irritability is even discussed as a key distinguishing criterion for bipolar subtypes in children and adolescents^8^. Irritability can present as ‘affective lability’, characterized by rapid mood shifts from pleasant to irritable or angry states, which is a significant predictor of bipolar episodes^9^. However, a 20-year prospective community-based study found that adolescent irritability could predict depression and anxiety but not bipolar disorder^10^. Therefore, the controversy about irritability and adolescent bipolar diagnosis, especially in differentiating bipolar disorder from chronic irritability, requires more evidence. The heritability of irritability is approximately 40–60%^7^, and addressing the causal association between irritability and bipolar disorder is crucial in resolving this inconsistency.

Irritability encompasses both behavioral and emotional components^7^. Smoking behaviors are often linked to emotional states, such as negative affect relief, difficulties in regulating emotional states, and fears of internal anxiety and bodily states^11^. Smoking is a major risk factor for disability-adjusted life years^12^. Additionally, individuals with mental disorders are more prone to tobacco dependence^13^. However, the relationship between irritability and smoking remains unclear, as does the interplay among irritability, smoking, and bipolar disorder.

Mendelian randomization (MR) study uses genetic variants as instrumental variables to explore the effects of exposures on outcomes, reducing potential biases caused by confounding and reverse causation^14^. The MR approach infers causal relationships between exposures and outcomes, overcoming the limitations of observational studies, founded on three core assumptions: strong association between instrumental variables and the exposure, no association between instrumental variables and confounding factors, and instrumental variables influencing the outcome solely through their association with the exposure. MR methodology has evolved to multivariable MR, which allows for equivalent analysis to mediation within the MR framework and can be used to estimate mediation effects^15^.

To our knowledge, there is limited evidence on the potential causal relationships among irritability, smoking, and bipolar disorder. Therefore, we hypothesize that irritability genetically increases the risk of smoking and bipolar disorder and can predict its occurrence. Smoking plays a mediating role in the relationship between irritability and bipolar disorder.

## METHODS

### Study design

A two-sample Mendelian randomization (MR) study design delineated a relationship between exposure and outcome. MR yields a genetically informed estimate of an exposure-outcome relation that is less likely to be biased due to unobserved confounding by treating single-nucleotide polymorphisms as a genetic instrument. Mendelian randomization relies on three core assumptions: 1) the relevance assumption – that the instrumental variables are strongly associated with the exposure; 2) the independence assumption – that the instrumental variables are not associated with any confounders of the exposure–outcome relationship; and 3) the exclusion restriction assumption – that the instrumental variables affect the outcome solely through their effect on the exposure, without alternative pathways. These assumptions form the foundation of the two-step Mendelian randomization (MR) mediation analysis employed in this study. Recent advances in MR methodology, such as multivariable MR, have further enabled the estimation of mediation effects within the MR framework by allowing for the simultaneous modeling of multiple exposures. In the first pairing, irritability serves as the exposure variable, and bipolar disorder is the outcome variable. In the second pairing, irritability is the exposure variable, and smoking status is the outcome variable. In the third pairing, smoking status is the exposure variable, and bipolar disorder is the outcome variable. This study employed a two-step MR analysis to investigate mediation analysis.

### Data sources and screen instrumental variables

The data sources were from the United Kingdom Biobank (UKB, containing Irritability, id: ukb-b-13745 used in this study), the European Bioinformatics Institute (EBI, containing Smoking, id: ebi-a-GCST90029014 used in this study), and the MRC Integrative Epidemiology Unit (IEU, containing bipolar disorder, id: ieu-b-41 used in this study). Detailed characteristics of the data sources are presented in Table 1. This study employed a Two-sample MR approach. We first extracted single nucleotide polymorphisms (SNPs) regarded as instrumental variables associated with traits with a p≤5×10^-8^ and a linkage disequilibrium threshold of r^2^ >0.001 within a 10000 kb window. The F-statistics (F=beta^2^/se^2^) indicating their predictive capability, were required to be >10. The F-statistics indicating the predictive strength of the instruments, were required to be >10. The F-values of the SNPs included in the analysis are provided in Supplementary file. Subsequently, these SNPs were harmonized and filtered using the Setiger filtering method.

**Table 1:** The characteristics of data sources used in the study

*Traits*	*Datasets*	*Unit*	*Ancestry*	*Sample size*	*Number of* *SNPs*	*Instrument* *variables*	*Author*	*Year*
Irritability	ukb-b-13745	SD	European	442169	9851867	29	B. Elsworth	2018
Smoking status	ebi-a-GCST90029014	SD	European	468170	11937425	39	P.R. Loh	2018
Bipolar disorder	ieu-b-41	NA	European	51710	13413244	91	E. Stahl	2019

SNPs: single nucleotide polymorphisms. SD: standard deviation. NA: not applicable.

### MR analysis and sensitivity analysis

The Mendelian randomization analysis was conducted using the Two Sample MR package in R Project for Statistical Computing (version 4.3.0). The primary method employed for assessing causal effects in Mendelian randomization was the inverse-variance-weighted (IVW) approach. However, as IVW can be sensitive to heterogeneity and horizontal pleiotropy, we performed additional sensitivity analyses using complementary MR methods, including MR-Egger regression, the weighted median method, and the weighted mode estimator, to evaluate the robustness of our findings and to account for potential violations of the MR assumptions. Heterogeneity among the Mendelian randomization results was evaluated using the Cochran-Q statistic test. To address potential horizontal pleiotropy, the MR-Egger intercept method was employed. To address potential horizontal pleiotropy, the MR-Egger intercept method was employed, which is based on the Instrument Strength Independent of Direct Effect (InSIDE) assumption. This assumption posits that the instrument-exposure associations are independent of the direct pleiotropic effects. The intercept term of MR-Egger provides an estimate of the average directional pleiotropy across instruments, allowing assessment and adjustment of potential pleiotropic bias. Additionally, a leave-one-out sensitivity test was conducted, which involved the sequential removal of single nucleotide polymorphisms (SNPs) to assess the impact of individual SNPs on the results.

### Mediation analysis

This study utilized the two-step MR to analyze the mediation effect^15,16^. First, univariable MR was used to investigate the potential causal relationships among the exposure, mediator, and outcome. If these conditions were met, the next stage involved conducting multivariable regressions to calculate the direct effect of the exposure on the outcome, with the mediator included as a covariate. The indirect effect was estimated by multiplying the regression estimates from the exposure-outcome regression and the mediator-outcome regression. The total effect was calculated as the sum of the direct and indirect effects. The proportion of the mediated effect was then determined by the ratio of the indirect effect to the total effect.

## RESULTS

We utilized single nucleotide polymorphisms (SNPs) as genetic instruments for irritability, smoking status, and bipolar disorder, identifying 29, 37, and 91 SNPs, respectively, after the screening process, as detailed in Table 1. Table 2 presents the results of univariable Mendelian randomization, demonstrating that the odds ratio (OR) of irritability for bipolar disorder was 3.13 (95% CI: 1.23–7.93; p=0.016). Furthermore, irritability exhibited a significant risk for smoking, with an odds ratio of 1.21 (95% CI: 1.07–1.37; p=0.002). Additionally, smoking serves as a significant risk factor for bipolar disorder (OR=2.00; 95% CI: 1.47–2.73; p<0.001). To further evaluate the robustness of the IVW estimates and account for potential horizontal pleiotropy, we conducted additional sensitivity analyses using MR-Egger, weighted median, and weighted mode approaches. As shown in Table 2, these complementary methods yielded results that were directionally consistent with those of the IVW method, providing additional support for the observed causal relationships. The convergence of findings across multiple analytical approaches strengthens the reliability of the associations identified.

**Table 2:** The results of the univariable two-sample Mendelian randomization study

*Exposure.id*	*Number of* *SNPs*	*Outcomes.id*	*OR (95% CI)*	*p*
**Irritability**	29	**Bipolar disorder**		
		Inverse variance weighted	3.13 (1.23–7.93)	0.016
		MR Egger	265 (1.09–64374)	0.056
		Weighted median	4.01 (1.96–13.45)	0.025
		Weighted mode	4.80 (0.36–63.85)	0.245
**Irritability**	37	**Smoking status**		
		Inverse variance weighted	1.23 (1.10–1.37)	0.000
		MR Egger	1.36 (0.72–2.58)	0.347
		Weighted median	1.30 (1.15–1.47)	0.000
		Weighted mode	1.45 (0.99–2.13)	0.064
**Smoking status**	91	**Bipolar disorder**		
		Inverse variance weighted	2.00 (1.47–2.73)	0.000
		MR Egger	1.84 (0.51–6.61)	0.351
		Weighted median	2.35 (1.45–3.82)	0.001
		Weighted mode	4.80 (1.30–17.72)	0.021

SNPs: single nucleotide polymorphisms.

The MR sensitivity analysis revealed no significant horizontal pleiotropy biases, as indicated by the MR-Egger intercept tests (all p>0.05), as shown in Table 3. Table 3 presents the results of Cochran’s Q statistic test, demonstrating heterogeneity in significance with the results of the irritability-smoking MR association. However, there was no significant heterogeneity in SNP effects in the other two groups (all p>0.05). Additionally, we performed MR-PRESSO (Mendelian Randomization Pleiotropy RESidual Sum and Outlier) analysis to further evaluate the presence of horizontal pleiotropy. The global test results showed no evidence of significant horizontal pleiotropy across all models (Global test: p=0.20, 0.33, and 0.64, respectively), supporting the robustness of our findings. Detailed MR-PRESSO results have been included in Table 3. The results of the leave-one-out method indicated that these causal relationships were not driven by single SNPs, as depicted in the Supplementary file.

**Table 3:** The analysis of heterogeneity and horizontal pleiotropy in the Mendelian randomization study

*Exposures*	*Outcome*	*Number of* *SNPs*	*Cochrane’s Q*				*Pleiotropy*		*Horizontal* *pleiotropy*	
			*MR-Egger*		*IVW*		*MR-Egger*		*MR-PRESSO*	
			*Q*	*p*	*Q*	*p*	*Egger intercept*	*p*	*Global test*	*p*
**Irritability**	Bipolar disorder	29	31.54	0.25	34.55	0.18	0.00	0.90	37.13	0.20
**Irritability**	Smoking status	39	108.03	0.00	108.46	0.00	0.00	0.70	84.45	0.33
**Smoking status**	Bipolar disorder	91	84.43	0.62	84.45	0.65	0.00	0.90	86.66	0.63

SNPs: single nucleotide polymorphisms. IVW: inverse variance weighted.

**Figure 1:**
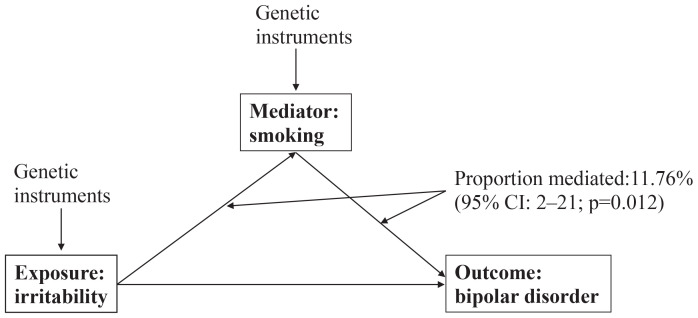
Mediations investigated in the present study

As illustrated in Figure 1, in the mediation analysis, the proportion of the effect of irritability on bipolar disorder mediated by smoking was 11.76% (95% CI: 2–21, p=0.012).

## DISCUSSION

The current investigation aimed to employ a two-step Mendelian randomization study to examine the genetic associations among irritability, smoking, and bipolar disorder and to analyze the mediation effect. The results revealed that irritability was significantly associated with bipolar disorder. Additionally, irritability was significantly associated with smoking, and smoking was significantly associated with bipolar disorder. Importantly, the association between irritability and bipolar disorder was partially mediated by smoking.

According to genetic evidence, irritability may contribute to an increased risk of bipolar disorder. Irritability often serves as an important criterion in diagnosing various adolescent psychiatric disorders, such as anxiety disorders, depression disorder, obsessive-compulsive disorder, attention-deficit/hyperactivity disorder, autism spectrum disorder, conduct disorder, and bipolar disorder, defined as an abnormal response to adversity or threat in individuals^7^. Irritability has been associated with significantly reduced activation in critical brain regions, including the left amygdala, the left and right striatum, the parietal cortex, and the posterior cingulate during tasks eliciting frustration^17^. Neuroimaging investigations of bipolar disorder have unveiled diminished cortical thickness in the anterior cingulate gyrus, alongside diminished amygdala volume^18^. Hence, there are similarities and differences in the abnormal brain regions of both irritability and bipolar disorder. Neurophysiological changes vary in different psychiatric disorders due to irritability, therefore, pinpointing the neurophysiological traits of irritability within bipolar disorder may furnish diagnostic specificity, differentiating it from other psychiatric conditions^19^. This diagnostic precision could potentially emerge as a pivotal biomarker for early bipolar disorder detection.

Our findings indicate that irritability is genetically associated with smoking through the MR study. Individuals with irritability tend to have an attentional bias toward threats in the early stages of emotional response, preceding their exaggerated reactions to stimuli, which aligns with the pathophysiological link to anxiety^20^. Negative consequence outcome expectancies regarding smoking were significantly related to anxiety sensitivity^11^. Neural dysfunction related to reward processing underlies clinically impairing irritability^21^. Smoking may increase reward sensitivity by alleviating anhedonia^22^. Therefore, smoking may represent externalizing behaviors as manifestations of irritability, in which individuals with emotion dysregulation, such as anxiety sensitivity, resort to smoking for relief.

Smoking is a genetically associated risk factor for bipolar disorder, consistent with the results of a previous study^14^ which illustrated that smoking initiation and lifetime smoking are identified as risk factors for bipolar disorder. The upregulation of nicotinic acetylcholine receptors in smokers may be attributed to this process after prolonged nicotine exposure^23^. Acetylcholine is an important neurotransmitter involved in the regulation of mood, cognition, and memory functions, and there may be dysfunctions in the acetylcholine system in patients with bipolar disorder^24^. Studies have found that drugs modulating acetylcholine receptors can induce manic episodes, suggesting that acetylcholine receptors may exhibit different functional patterns or expression levels in the brains of patients with bipolar disorder^25^.

The partial mediation of smoking in the genetic association between irritability and bipolar disorder has been demonstrated in this study. Anxiety traits may contribute to this mediation. At least half of bipolar disorder patients may experience anxiety in their lives^9,26^. Anxiety often precedes the onset of bipolar disorder in comorbid cases^27^. Anxiety symptoms are also a risk factor for bipolar disorder in the absence of comorbidity^9^. The inherent emotional dysregulation in irritable individuals can manifest as anxiety and externalize as heightened threat sensitivity^20^. Meanwhile, irritability is regarded as an abnormal response to frustration, arising when goal attainment is obstructed or expected rewards are withheld^28^. Smoking can relieve this high anxiety vulnerability. Nicotine, the primary psychoactive ingredient in cigarettes, interacts with neurotransmitters^29^, temporarily alleviating irritability and providing a nicotine-dependent reward for individuals with bipolar disorder. In addition, irritability represents an overreaction to stress. Smoking can activate the hypothalamic-pituitary-adrenal axis^30^, providing temporary emotional stability. However, smoking leads to chronic inflammation and neurotoxicity, thereby exacerbating the progression of bipolar disorder.

### Limitations

The current GWAS data are limited to European populations, and the applicability of our conclusions in other ancestries needs further verification. In this study, examining the relationship of irritability being associated with smoking, indicated potential heterogeneity based on the Q-statistic results, though no outliers were observed in the leave-one-out test. We hypothesize that the observed heterogeneity may be due to the following reasons: measurement errors, which could be present in other variables. Multiple pathogenic mechanisms may also be one of the reasons. Smoking may be influenced by various factors such as social environment and peer pressure, which were not accounted for in the analysis, thus contributing to the heterogeneity in the results. In addition, our study data are from Europeans, which makes it difficult to generalize to other populations. Our data have already been collected and are readymade data, so it is difficult to generalize the results across other exposure periods/timings, and across other levels of exposure

Based on our results, identifying the neurophysiological characteristics of irritability specific to bipolar disorder, distinct from those associated with other psychiatric conditions, may enable early screening, diagnosis, and differentiation of bipolar disorder based on these neurophysiological markers. In terms of treatment, stimulants and selective serotonin reuptake inhibitors (SSRIs) can reduce irritability, particularly when they underlie depressive and anxious frameworks. However, these medications are contraindicated for bipolar disorder, as antidepressants may be a significant factor in the transition from a diagnosis of depression to bipolar disorder. Therefore, during the prodromal phase or early clinical stages of bipolar disorder, alternative treatment methods and strategies can help manage irritability without the risks associated with stimulants and SSRIs. These alternatives include parent training and cognitive-behavioral therapy^7^. Evidence suggests that individuals with bipolar disorder are two to three times more likely to smoke and face greater challenges quitting, partly due to underlying emotional dysregulation^31,32^. These findings highlight the need for further research into the mechanisms linking smoking, irritability, and bipolar disorder. As a modifiable risk factor, smoking cessation can be promoted through preventive measures and public health campaigns that emphasize the importance of a supportive environment for mental health, thereby improving overall outcomes for individuals with bipolar disorder.

## CONCLUSIONS

This two-step Mendelian randomization study suggests a potential causal pathway from irritability to bipolar disorder, with smoking acting as a partial mediator. Irritability was genetically associated with both smoking and bipolar disorder, and smoking accounted for approximately 11.76% of the total effect. As a modifiable risk factor, smoking may represent a target for early intervention in individuals with heightened irritability. These findings highlight the importance of addressing emotional dysregulation and smoking behaviors to mitigate bipolar disorder.

## Supplementary Material



## Data Availability

The data supporting this research are available from the following sources: https://gwas.mrcieu.ac.uk/ (id: ukb-b-13745; ebi-aGCST90029014; ieu-b-41, respectively).
