# Supplementary material for: The causal mediating effect of smoking on the relationship between irritability and bipolar disorder: A two-step Mendelian randomization study
Source: Tob Induc Dis. 2025 Nov 7;23:10.18332/tid/209615. doi: 10.18332/tid/209615 (PMC12598469; doi:10.18332/tid/209615)
Supplement: Supplementary file 1 [file TID-23-174-s1.pdf]

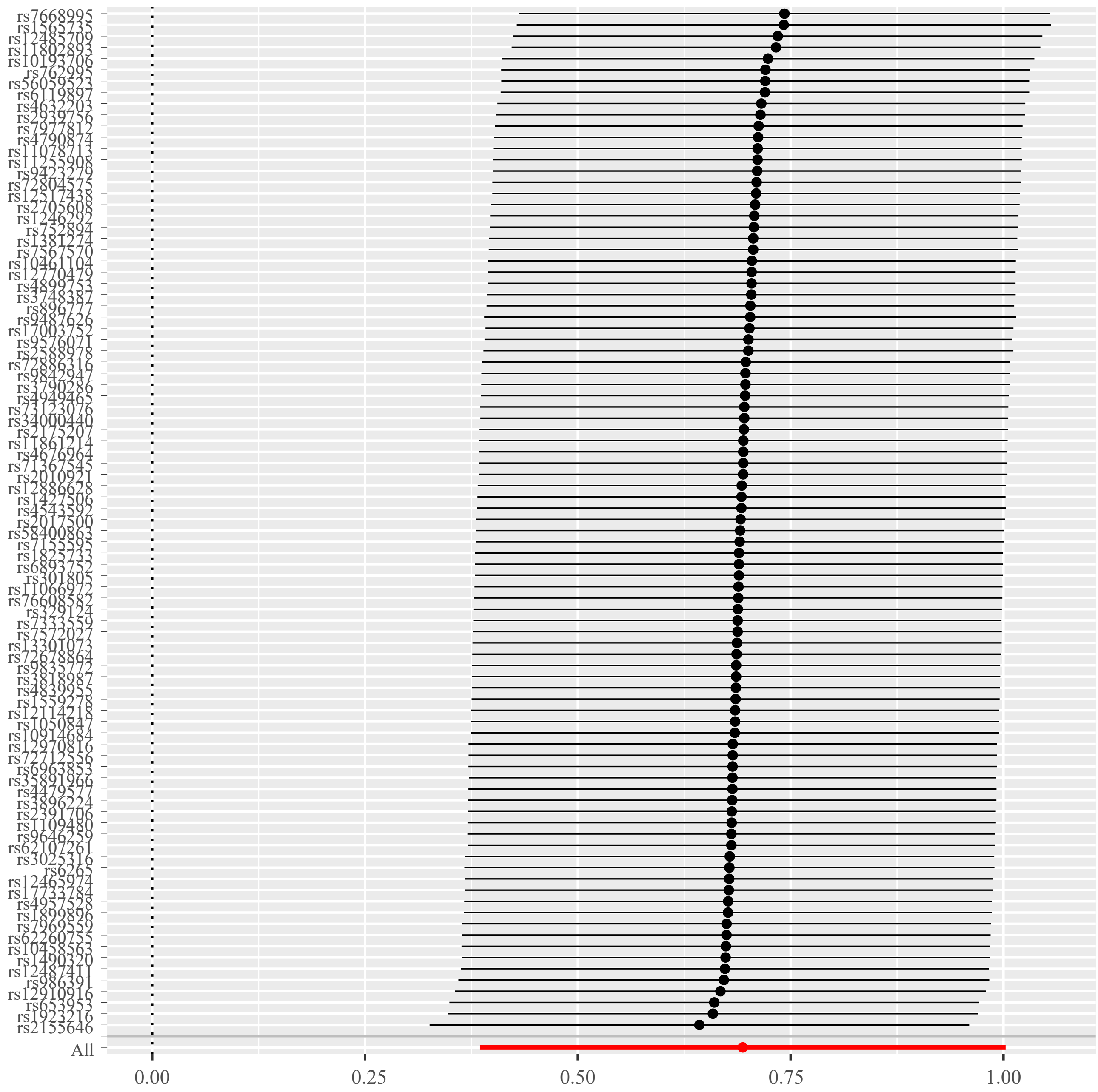

Supplementary Figure 1.1 MR leave-one-out sensitivity analysis for
'Smoking status || id:ebi-a-GCST90029014' on 'bipolar disorder || id:ieu-b-41'

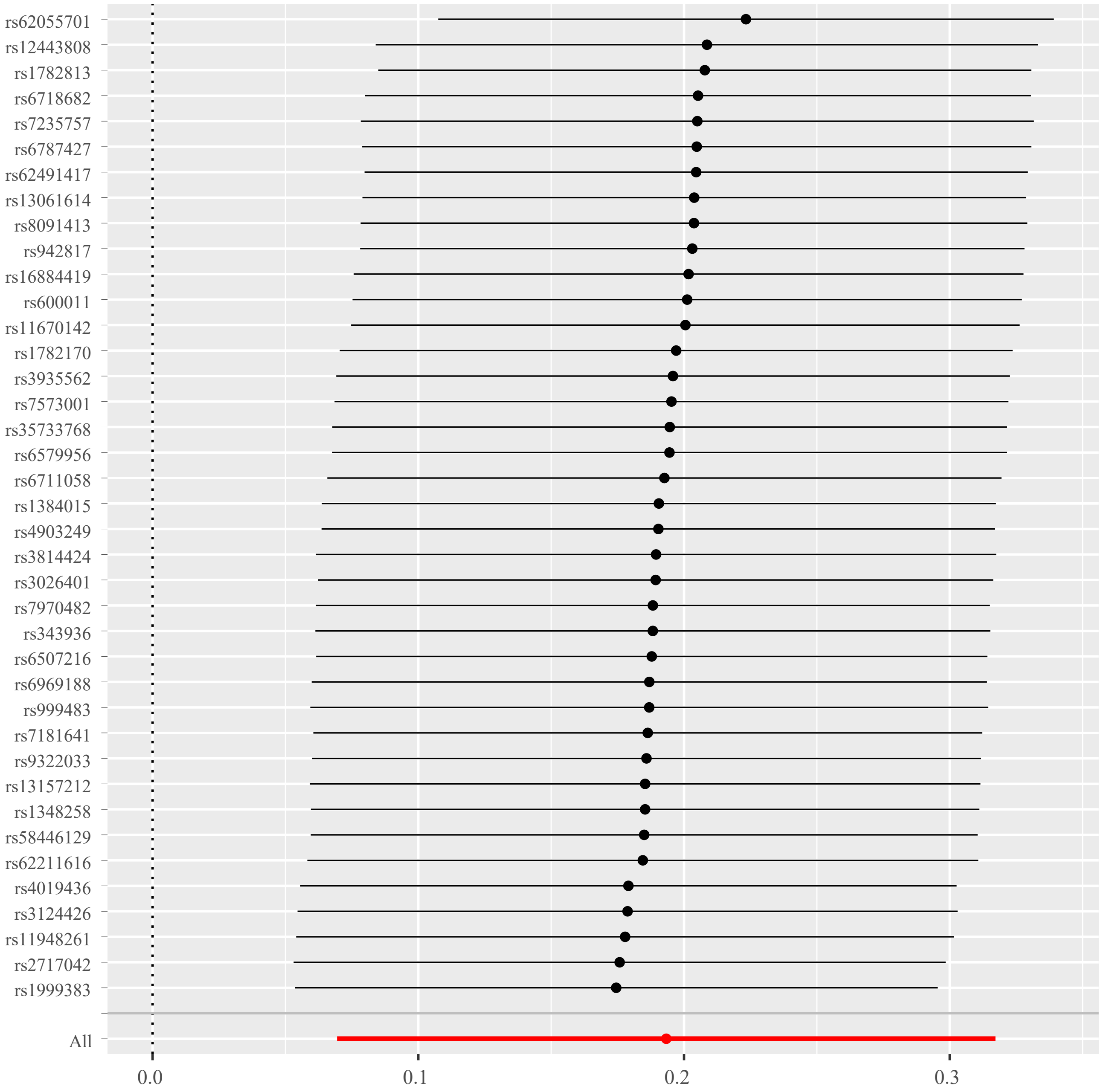

Supplementary Figure 1.2 MR leave-one-out sensitivity analysis for
'Irritability || id:ukb-b-13745' on 'Smoking status || id:ebi-a-GCST90029014'

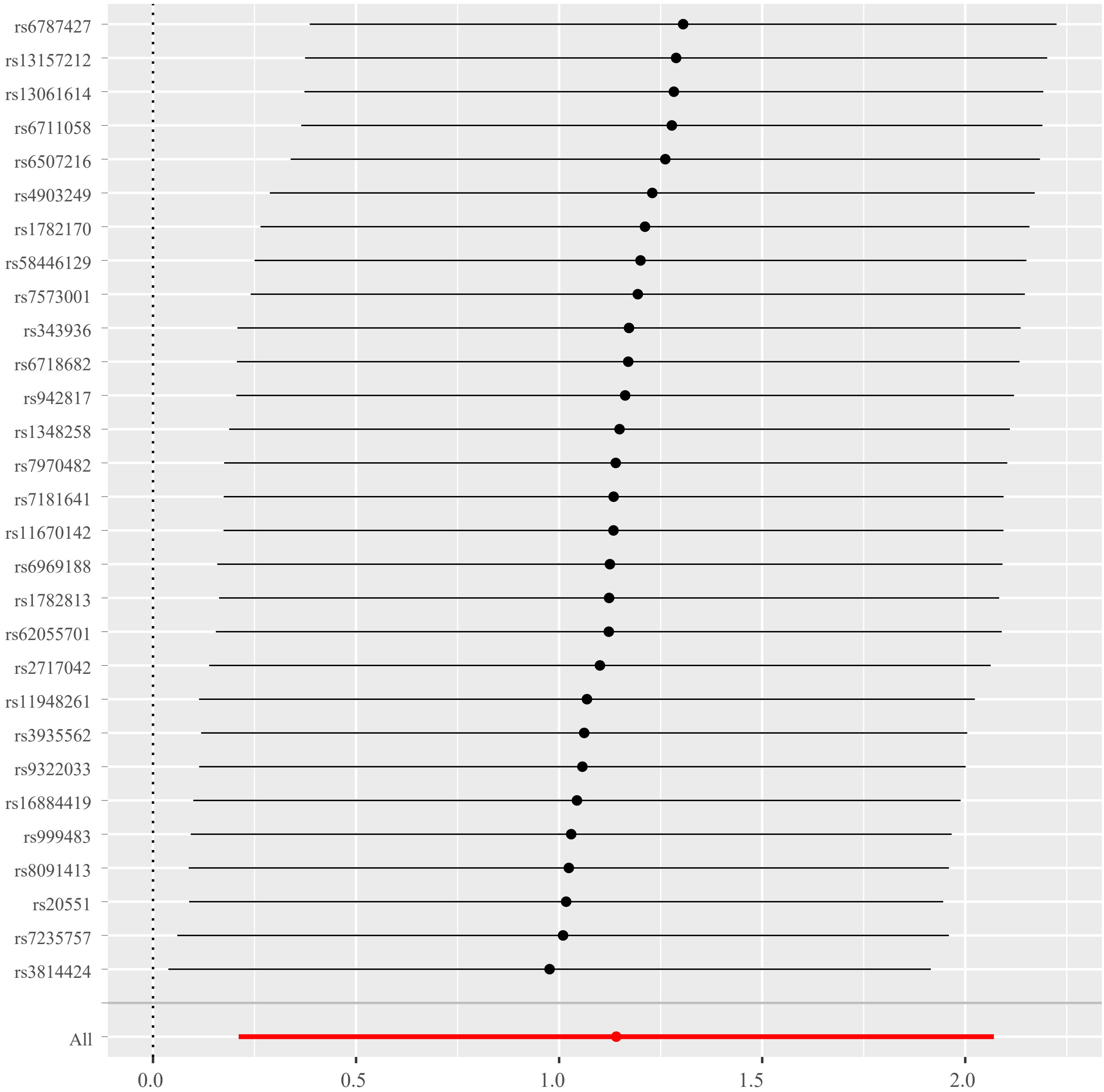

Supplementary Figure 1.3 MR leave-one-out sensitivity analysis for 'Irritability || id:ukb-b-13745' on 'bipolar disorder || id:ieu-b-41'

supplementary materials 2

SNP	effect_allele.exposure	other_allele.exposure	effect_allele.outcome	other_allele.outcome	beta.exposure	beta.outcome	eaf.exposure	eaf.outcome	remove	palindromic	ambiguous	id.outcome	chr	pos	se.outcome	samplesize.outcome	pval.outcome
rs11670142	G	T	G	T	0.00537733	0.007397	0.394986	0.395213	FALSE	FALSE	FALSE	ieu-b-41	19	49650471	0.0137	51710	0.588499367
rs11948261	A	T	A	T	0.00749452	0.022202	0.214042	0.211181	FALSE	TRUE	FALSE	ieu-b-41	5	107753691	0.0164	51710	0.175099941
rs13061614	C	T	C	T	0.0055326	-0.019303	0.408832	0.390213	FALSE	FALSE	FALSE	ieu-b-41	3	35694457	0.0137	51710	0.160000006
rs13157212	C	A	C	A	-0.00864971	0.027001	0.134613	0.133361	FALSE	FALSE	FALSE	ieu-b-41	5	46055494	0.0202	51710	0.179899932
rs1348258	A	G	A	G	-0.00568548	-0.005002	0.634266	0.648394	FALSE	FALSE	FALSE	ieu-b-41	15	87980070	14	51710	0.722099426
rs16884419	A	G	A	G	0.00691058	0.025902	0.237483	0.244755	FALSE	FALSE	FALSE	ieu-b-41	8	89579649	0.0156	51710	0.097310566
rs1782170	C	A	C	A	0.00596707	-0.007998	0.724225	0.732606	FALSE	FALSE	FALSE	ieu-b-41	14	41674903	0.0153	51710	0.601299718
rs1782813	C	T	C	T	0.00723743	0.012397	0.834799	0.833574	FALSE	FALSE	FALSE	ieu-b-41	1	98499843	0.0179	51710	0.489299757
rs20551	G	A	G	A	0.00631102	0.029903	0.288044	0.284542	FALSE	FALSE	FALSE	ieu-b-41	22	41548008	0.0148	51710	0.043430017
rs2717042	A	C	A	C	0.00630191	0.013597	0.625849	0.626181	FALSE	FALSE	FALSE	ieu-b-41	2	58177082	0.0138	51710	0.323600363
rs343936	C	T	C	T	-0.00831085	-0.003095	0.167482	167	FALSE	FALSE	FALSE	ieu-b-41	2	44992550	0.0179	51710	0.863699961
rs3814424	T	C	T	C	0.0100905	0.039403	0.157588	0.160755	FALSE	FALSE	FALSE	ieu-b-41	5	87968953	0.0182	51710	0.030150186
rs3935562	A	G	A	G	0.00525339	0.020596	0.490421	0.491181	FALSE	FALSE	FALSE	ieu-b-41	9	121275721	0.0135	51710	0.125800104
rs4903249	T	C	T	C	0.00539923	-0.009303	0.489003	0.495394	FALSE	FALSE	FALSE	ieu-b-41	14	75083881	0.0133	51710	0.485499738
rs58446129	T	C	T	C	0.00746415	-0.007397	0.146099	149	FALSE	FALSE	FALSE	ieu-b-41	13	66582410	0.0193	48569	0.702599765
rs62055701	A	G	A	G	0.00797808	0.012304	0.222601	0.214181	FALSE	FALSE	FALSE	ieu-b-41	17	43758787	0.0163	51710	0.450000504
rs6507216	T	G	T	G	0.00561016	-0.016404	0.34275	0.339394	FALSE	FALSE	FALSE	ieu-b-41	18	35195719	0.0141	51710	0.245499719
rs6711058	A	G	A	G	0.00605901	-0.020499	0.735317	0.729819	FALSE	FALSE	FALSE	ieu-b-41	2	205116051	0.0151	51710	0.173700073
rs6718682	T	C	T	C	-0.00692323	-0.002704	0.275458	0.264213	FALSE	FALSE	FALSE	ieu-b-41	2	122663627	0.0152	51710	0.857200074
rs6787427	C	T	C	T	0.00743874	-0.019096	0.260465	0.257426	FALSE	FALSE	FALSE	ieu-b-41	3	85556518	0.0154	51710	0.214699978
rs6969188	A	G	A	G	0.0065984	0.009901	0.450912	0.430213	FALSE	FALSE	FALSE	ieu-b-41	7	114160742	0.0138	51710	0.471099887
rs7181641	G	A	G	A	0.00741224	0.010101	0.147497	149	FALSE	FALSE	FALSE	ieu-b-41	15	61744669	0.0189	51710	0.591599775
rs7235757	A	G	A	G	0.00789323	0.027099	0.312851	0.306542	FALSE	FALSE	FALSE	ieu-b-41	18	53067954	0.0145	51710	0.060869537
rs7573001	C	G	C	G	-0.00548406	0.003703	0.38134	0.39	FALSE	TRUE	FALSE	ieu-b-41	2	198929896	0.0139	51710	0.790099888
rs7970482	A	G	A	G	0.00638429	0.007502	0.311411	0.314361	FALSE	FALSE	FALSE	ieu-b-41	12	109862335	0.0144	51710	0.602599823
rs8091413	G	C	G	C	-0.00676219	-0.028801	0.744208	0.741606	FALSE	TRUE	FALSE	ieu-b-41	18	63549860	0.0152	51710	0.057729793
rs9322033	T	C	T	C	0.00642006	0.0248	0.224942	0.229181	FALSE	FALSE	FALSE	ieu-b-41	6	100852016	0.0159	51710	0.117999998
rs942817	C	G	C	G	0.00546792	0.001599	0.612072	0.610181	FALSE	TRUE	FALSE	ieu-b-41	1	2413634	0.0147	51710	0.914899942
rs999483	G	T	G	T	0.00825844	0.035099	0.249215	0.222181	FALSE	FALSE	FALSE	ieu-b-41	9	135301389	19	51710	0.064159357

<table>
<tr><th>SNP</th><th>outcome</th><th>originalname.outcome</th><th>outcome.deprecated</th><th>mr_keep.outcome</th><th>data_source.outcome</th></tr>
<tr><td>rs11670142</td><td>bipolar disorder || id:ieu-b-41</td><td>bipolar disorder</td><td>bipolar disorder || ||</td><td>TRUE</td><td>MedicineITLab</td></tr>
<tr><td>rs11948261</td><td>bipolar disorder || id:ieu-b-41</td><td>bipolar disorder</td><td>bipolar disorder || ||</td><td>TRUE</td><td>MedicineITLab</td></tr>
<tr><td>rs13061614</td><td>bipolar disorder || id:ieu-b-41</td><td>bipolar disorder</td><td>bipolar disorder || ||</td><td>TRUE</td><td>MedicineITLab</td></tr>
<tr><td>rs13157212</td><td>bipolar disorder || id:ieu-b-41</td><td>bipolar disorder</td><td>bipolar disorder || ||</td><td>TRUE</td><td>MedicineITLab</td></tr>
<tr><td>rs1348258</td><td>bipolar disorder || id:ieu-b-41</td><td>bipolar disorder</td><td>bipolar disorder || ||</td><td>TRUE</td><td>MedicineITLab</td></tr>
<tr><td>rs16884419</td><td>bipolar disorder || id:ieu-b-41</td><td>bipolar disorder</td><td>bipolar disorder || ||</td><td>TRUE</td><td>MedicineITLab</td></tr>
<tr><td>rs1782170</td><td>bipolar disorder || id:ieu-b-41</td><td>bipolar disorder</td><td>bipolar disorder || ||</td><td>TRUE</td><td>MedicineITLab</td></tr>
<tr><td>rs1782813</td><td>bipolar disorder || id:ieu-b-41</td><td>bipolar disorder</td><td>bipolar disorder || ||</td><td>TRUE</td><td>MedicineITLab</td></tr>
<tr><td>rs20551</td><td>bipolar disorder || id:ieu-b-41</td><td>bipolar disorder</td><td>bipolar disorder || ||</td><td>TRUE</td><td>MedicineITLab</td></tr>
<tr><td>rs2717042</td><td>bipolar disorder || id:ieu-b-41</td><td>bipolar disorder</td><td>bipolar disorder || ||</td><td>TRUE</td><td>MedicineITLab</td></tr>
<tr><td>rs343936</td><td>bipolar disorder || id:ieu-b-41</td><td>bipolar disorder</td><td>bipolar disorder || ||</td><td>TRUE</td><td>MedicineITLab</td></tr>
<tr><td>rs3814424</td><td>bipolar disorder || id:ieu-b-41</td><td>bipolar disorder</td><td>bipolar disorder || ||</td><td>TRUE</td><td>MedicineITLab</td></tr>
<tr><td>rs3935562</td><td>bipolar disorder || id:ieu-b-41</td><td>bipolar disorder</td><td>bipolar disorder || ||</td><td>TRUE</td><td>MedicineITLab</td></tr>
<tr><td>rs4903249</td><td>bipolar disorder || id:ieu-b-41</td><td>bipolar disorder</td><td>bipolar disorder || ||</td><td>TRUE</td><td>MedicineITLab</td></tr>
<tr><td>rs58446129</td><td>bipolar disorder || id:ieu-b-41</td><td>bipolar disorder</td><td>bipolar disorder || ||</td><td>TRUE</td><td>MedicineITLab</td></tr>
<tr><td>rs62055701</td><td>bipolar disorder || id:ieu-b-41</td><td>bipolar disorder</td><td>bipolar disorder || ||</td><td>TRUE</td><td>MedicineITLab</td></tr>
<tr><td>rs6507216</td><td>bipolar disorder || id:ieu-b-41</td><td>bipolar disorder</td><td>bipolar disorder || ||</td><td>TRUE</td><td>MedicineITLab</td></tr>
<tr><td>rs6711058</td><td>bipolar disorder || id:ieu-b-41</td><td>bipolar disorder</td><td>bipolar disorder || ||</td><td>TRUE</td><td>MedicineITLab</td></tr>
<tr><td>rs6718682</td><td>bipolar disorder || id:ieu-b-41</td><td>bipolar disorder</td><td>bipolar disorder || ||</td><td>TRUE</td><td>MedicineITLab</td></tr>
<tr><td>rs6787427</td><td>bipolar disorder || id:ieu-b-41</td><td>bipolar disorder</td><td>bipolar disorder || ||</td><td>TRUE</td><td>MedicineITLab</td></tr>
<tr><td>rs6969188</td><td>bipolar disorder || id:ieu-b-41</td><td>bipolar disorder</td><td>bipolar disorder || ||</td><td>TRUE</td><td>MedicineITLab</td></tr>
<tr><td>rs7181641</td><td>bipolar disorder || id:ieu-b-41</td><td>bipolar disorder</td><td>bipolar disorder || ||</td><td>TRUE</td><td>MedicineITLab</td></tr>
<tr><td>rs7235757</td><td>bipolar disorder || id:ieu-b-41</td><td>bipolar disorder</td><td>bipolar disorder || ||</td><td>TRUE</td><td>MedicineITLab</td></tr>
<tr><td>rs7573001</td><td>bipolar disorder || id:ieu-b-41</td><td>bipolar disorder</td><td>bipolar disorder || ||</td><td>TRUE</td><td>MedicineITLab</td></tr>
<tr><td>rs7970482</td><td>bipolar disorder || id:ieu-b-41</td><td>bipolar disorder</td><td>bipolar disorder || ||</td><td>TRUE</td><td>MedicineITLab</td></tr>
<tr><td>rs8091413</td><td>bipolar disorder || id:ieu-b-41</td><td>bipolar disorder</td><td>bipolar disorder || ||</td><td>TRUE</td><td>MedicineITLab</td></tr>
<tr><td>rs9322033</td><td>bipolar disorder || id:ieu-b-41</td><td>bipolar disorder</td><td>bipolar disorder || ||</td><td>TRUE</td><td>MedicineITLab</td></tr>
<tr><td>rs942817</td><td>bipolar disorder || id:ieu-b-41</td><td>bipolar disorder</td><td>bipolar disorder || ||</td><td>TRUE</td><td>MedicineITLab</td></tr>
<tr><td>rs999483</td><td>bipolar disorder || id:ieu-b-41</td><td>bipolar disorder</td><td>bipolar disorder || ||</td><td>TRUE</td><td>MedicineITLab</td></tr>
</table>

<table>
<tr><th>SNP</th><th>pval.exposure</th><th>pos.exposure</th><th>chr.exposure</th><th>se.exposure</th><th>samplesize.exposure</th><th>id.exposure</th><th>exposure</th><th>mr_keep.exposure</th><th>pval_origin.exposure</th><th>data_source.exposure</th></tr>
<tr><td>rs11670142</td><td>3.50E-08</td><td>49650471</td><td>19</td><td>0.000975364</td><td>442169</td><td>ukb-b-13745</td><td>Irritability || id:ukb-b-13745</td><td>TRUE</td><td>reported</td><td>igd</td></tr>
<tr><td>rs11948261</td><td>1.20E-10</td><td>107753691</td><td>5</td><td>0.00116477</td><td>442169</td><td>ukb-b-13745</td><td>Irritability || id:ukb-b-13745</td><td>TRUE</td><td>reported</td><td>igd</td></tr>
<tr><td>rs13061614</td><td>1.20E-08</td><td>35694457</td><td>3</td><td>0.00097168</td><td>442169</td><td>ukb-b-13745</td><td>Irritability || id:ukb-b-13745</td><td>TRUE</td><td>reported</td><td>igd</td></tr>
<tr><td>rs13157212</td><td>6.40E-10</td><td>46055494</td><td>5</td><td>0.00139966</td><td>442169</td><td>ukb-b-13745</td><td>Irritability || id:ukb-b-13745</td><td>TRUE</td><td>reported</td><td>igd</td></tr>
<tr><td>rs1348258</td><td>1.00E-08</td><td>87980070</td><td>15</td><td>0.000992822</td><td>442169</td><td>ukb-b-13745</td><td>Irritability || id:ukb-b-13745</td><td>TRUE</td><td>reported</td><td>igd</td></tr>
<tr><td>rs16884419</td><td>7.40E-10</td><td>89579649</td><td>8</td><td>0.00112244</td><td>442169</td><td>ukb-b-13745</td><td>Irritability || id:ukb-b-13745</td><td>TRUE</td><td>reported</td><td>igd</td></tr>
<tr><td>rs1782170</td><td>2.50E-08</td><td>41674903</td><td>14</td><td>0.0010713</td><td>442169</td><td>ukb-b-13745</td><td>Irritability || id:ukb-b-13745</td><td>TRUE</td><td>reported</td><td>igd</td></tr>
<tr><td>rs1782813</td><td>1.70E-08</td><td>98499843</td><td>1</td><td>0.00128238</td><td>442169</td><td>ukb-b-13745</td><td>Irritability || id:ukb-b-13745</td><td>TRUE</td><td>reported</td><td>igd</td></tr>
<tr><td>rs20551</td><td>2.10E-09</td><td>41548008</td><td>22</td><td>0.00105324</td><td>442169</td><td>ukb-b-13745</td><td>Irritability || id:ukb-b-13745</td><td>TRUE</td><td>reported</td><td>igd</td></tr>
<tr><td>rs2717042</td><td>1.60E-10</td><td>58177082</td><td>2</td><td>0.000985371</td><td>442169</td><td>ukb-b-13745</td><td>Irritability || id:ukb-b-13745</td><td>TRUE</td><td>reported</td><td>igd</td></tr>
<tr><td>rs343936</td><td>9.00E-11</td><td>44992550</td><td>2</td><td>0.00128194</td><td>442169</td><td>ukb-b-13745</td><td>Irritability || id:ukb-b-13745</td><td>TRUE</td><td>reported</td><td>igd</td></tr>
<tr><td>rs3814424</td><td>1.30E-14</td><td>87968953</td><td>5</td><td>0.00130881</td><td>442169</td><td>ukb-b-13745</td><td>Irritability || id:ukb-b-13745</td><td>TRUE</td><td>reported</td><td>igd</td></tr>
<tr><td>rs3935562</td><td>3.70E-08</td><td>121275721</td><td>9</td><td>0.000954195</td><td>442169</td><td>ukb-b-13745</td><td>Irritability || id:ukb-b-13745</td><td>TRUE</td><td>reported</td><td>igd</td></tr>
<tr><td>rs4903249</td><td>1.40E-08</td><td>75083881</td><td>14</td><td>0.000952656</td><td>442169</td><td>ukb-b-13745</td><td>Irritability || id:ukb-b-13745</td><td>TRUE</td><td>reported</td><td>igd</td></tr>
<tr><td>rs58446129</td><td>3.20E-08</td><td>66582410</td><td>13</td><td>0.00134995</td><td>442169</td><td>ukb-b-13745</td><td>Irritability || id:ukb-b-13745</td><td>TRUE</td><td>reported</td><td>igd</td></tr>
<tr><td>rs62055701</td><td>3.60E-12</td><td>43758787</td><td>17</td><td>0.00114749</td><td>442169</td><td>ukb-b-13745</td><td>Irritability || id:ukb-b-13745</td><td>TRUE</td><td>reported</td><td>igd</td></tr>
<tr><td>rs6507216</td><td>2.40E-08</td><td>35195719</td><td>18</td><td>0.00100569</td><td>442169</td><td>ukb-b-13745</td><td>Irritability || id:ukb-b-13745</td><td>TRUE</td><td>reported</td><td>igd</td></tr>
<tr><td>rs6711058</td><td>3.20E-08</td><td>205116051</td><td>2</td><td>0.00109579</td><td>442169</td><td>ukb-b-13745</td><td>Irritability || id:ukb-b-13745</td><td>TRUE</td><td>reported</td><td>igd</td></tr>
<tr><td>rs6718682</td><td>9.70E-11</td><td>122663627</td><td>2</td><td>0.00106988</td><td>442169</td><td>ukb-b-13745</td><td>Irritability || id:ukb-b-13745</td><td>TRUE</td><td>reported</td><td>igd</td></tr>
<tr><td>rs6787427</td><td>6.40E-12</td><td>85556518</td><td>3</td><td>0.00108276</td><td>442169</td><td>ukb-b-13745</td><td>Irritability || id:ukb-b-13745</td><td>TRUE</td><td>reported</td><td>igd</td></tr>
<tr><td>rs6969188</td><td>6.70E-12</td><td>114160742</td><td>7</td><td>0.000961371</td><td>442169</td><td>ukb-b-13745</td><td>Irritability || id:ukb-b-13745</td><td>TRUE</td><td>reported</td><td>igd</td></tr>
<tr><td>rs7181641</td><td>4.20E-08</td><td>61744669</td><td>15</td><td>0.00135225</td><td>442169</td><td>ukb-b-13745</td><td>Irritability || id:ukb-b-13745</td><td>TRUE</td><td>reported</td><td>igd</td></tr>
<tr><td>rs7235757</td><td>2.40E-14</td><td>53067954</td><td>18</td><td>0.00103515</td><td>442169</td><td>ukb-b-13745</td><td>Irritability || id:ukb-b-13745</td><td>TRUE</td><td>reported</td><td>igd</td></tr>
<tr><td>rs7573001</td><td>2.70E-08</td><td>198929896</td><td>2</td><td>0.000986007</td><td>442169</td><td>ukb-b-13745</td><td>Irritability || id:ukb-b-13745</td><td>TRUE</td><td>reported</td><td>igd</td></tr>
<tr><td>rs7970482</td><td>5.60E-10</td><td>109862335</td><td>12</td><td>0.00102952</td><td>442169</td><td>ukb-b-13745</td><td>Irritability || id:ukb-b-13745</td><td>TRUE</td><td>reported</td><td>igd</td></tr>
<tr><td>rs8091413</td><td>6.40E-10</td><td>63549860</td><td>18</td><td>0.00109421</td><td>442169</td><td>ukb-b-13745</td><td>Irritability || id:ukb-b-13745</td><td>TRUE</td><td>reported</td><td>igd</td></tr>
<tr><td>rs9322033</td><td>1.80E-08</td><td>100852016</td><td>6</td><td>0.00114038</td><td>442169</td><td>ukb-b-13745</td><td>Irritability || id:ukb-b-13745</td><td>TRUE</td><td>reported</td><td>igd</td></tr>
<tr><td>rs942817</td><td>2.40E-08</td><td>2413634</td><td>1</td><td>0.000979456</td><td>442169</td><td>ukb-b-13745</td><td>Irritability || id:ukb-b-13745</td><td>TRUE</td><td>reported</td><td>igd</td></tr>
<tr><td>rs999483</td><td>6.40E-14</td><td>135301389</td><td>9</td><td>0.00110109</td><td>442169</td><td>ukb-b-13745</td><td>Irritability || id:ukb-b-13745</td><td>TRUE</td><td>reported</td><td>igd</td></tr>
</table>

SNP	action	mr_keep	units.outcome	units.exposure	rsq.exposure	effective_n.exposure	rsq.outcome	effective_n.outcome	steiger_dir	steiger_pval	R2	F	FMean	F_pow_beta_se
rs11670142	2	TRUE			6.87E-05	442169	5.64E-06	51710	TRUE	0.203022598	1.38E-05	6.110866984	7.710421139	30.39484512
rs11948261	2	TRUE			9.36E-05	442169	3.54E-05	51710	TRUE	0.423141094	1.89E-05	8.356232499	7.710421139	41.40066272
rs13061614	2	TRUE			7.33E-05	442169	3.84E-05	51710	TRUE	0.610633664	1.48E-05	6.542399988	7.710421139	32.41992585
rs13157212	2	TRUE			8.64E-05	442169	3.46E-05	51710	TRUE	0.462445402	1.74E-05	7.707692093	7.710421139	38.19073278
rs1348258	2	TRUE			7.42E-05	442169	2.47E-06	51710	TRUE	0.129808094	1.50E-05	6.631226703	7.710421139	32.79378069
rs16884419	2	TRUE			8.57E-05	442169	5.33E-05	51710	TRUE	0.673705243	1.73E-05	7.647771244	7.710421139	37.90554347
rs1782170	2	TRUE			7.02E-05	442169	5.28E-06	51710	TRUE	0.191003347	1.42E-05	6.288878009	7.710421139	31.02416171
rs1782813	2	TRUE			7.20E-05	442169	9.28E-06	51710	TRUE	0.241673888	1.44E-05	6.3883094	7.710421139	31.85189536
rs20551	2	TRUE			8.12E-05	442169	7.89E-05	51710	TRUE	0.978410037	1.63E-05	7.223281129	7.710421139	35.90413136
rs2717042	2	TRUE			9.25E-05	442169	1.88E-05	51710	TRUE	0.255509801	1.86E-05	8.22404099	7.710421139	40.90202783
rs343936	2	TRUE			9.50E-05	442169	5.78E-07	51710	TRUE	0.053104619	1.93E-05	8.516817599	7.710421139	42.02962257
rs3814424	2	TRUE			0.000134409	442169	9.06E-05	51710	TRUE	0.655545382	2.70E-05	11.95366623	7.710421139	59.43909171
rs3935562	2	TRUE			6.85E-05	442169	4.50E-05	51710	TRUE	0.735455201	1.38E-05	6.099330689	7.710421139	30.31133142
rs4903249	2	TRUE			7.26E-05	442169	9.46E-06	51710	TRUE	0.241203676	1.46E-05	6.441932654	7.710421139	32.12117639
rs58446129	2	TRUE			6.91E-05	442169	3.02E-06	48569	TRUE	0.168943772	1.39E-05	6.146640911	7.710421139	30.57210556
rs62055701	2	TRUE			0.000109311	442169	1.10E-05	51710	TRUE	0.124689677	2.20E-05	9.740780394	7.710421139	48.33914391
rs6507216	2	TRUE			7.04E-05	442169	2.62E-05	51710	TRUE	0.481322094	1.42E-05	6.270194809	7.710421139	31.11875628
rs6711058	2	TRUE			6.91E-05	442169	3.56E-05	51710	TRUE	0.613835914	1.43E-05	6.318682338	7.710421139	30.57374739
rs6718682	2	TRUE			9.47E-05	442169	6.12E-07	51710	TRUE	0.054170829	1.91E-05	8.459819691	7.710421139	41.87428277
rs6787427	2	TRUE			0.000106734	442169	2.97E-05	51710	TRUE	0.293876968	2.13E-05	9.426108743	7.710421139	47.19916896
rs6969188	2	TRUE			0.000106528	442169	9.95E-06	51710	TRUE	0.123091852	2.16E-05	9.533156229	7.710421139	47.10806263
rs7181641	2	TRUE			6.79E-05	442169	5.52E-06	51710	TRUE	0.204831826	1.38E-05	6.109425483	7.710421139	30.04588256
rs7235757	2	TRUE			0.00013148	442169	6.75E-05	51710	TRUE	0.484613226	2.68E-05	11.84475108	7.710421139	58.14373721
rs7573001	2	TRUE			7.00E-05	442169	1.37E-06	51710	TRUE	0.121727608	1.42E-05	6.274675638	7.710421139	30.93459247
rs7970482	2	TRUE			8.70E-05	442169	5.25E-06	51710	TRUE	0.130139042	1.75E-05	7.729353258	7.710421139	38.45524978
rs8091413	2	TRUE			8.64E-05	442169	6.94E-05	51710	TRUE	0.836177824	1.74E-05	7.698035574	7.710421139	38.19208598
rs9322033	2	TRUE			7.17E-05	442169	4.70E-05	51710	TRUE	0.729511308	1.44E-05	6.354852776	7.710421139	31.69414129
rs942817	2	TRUE			7.05E-05	442169	2.29E-07	51710	TRUE	0.088490692	1.42E-05	6.277987296	7.710421139	31.16552461
rs999483	2	TRUE			0.000127206	442169	6.60E-05	51710	TRUE	0.497207953	2.55E-05	11.28530259	7.710421139	56.25361012

© 2025 Hu Q. et al.
